# Familial Hypercholesterolemia: The Most Frequent Cholesterol Metabolism Disorder Caused Disease

**DOI:** 10.3390/ijms19113426

**Published:** 2018-11-01

**Authors:** Asier Benito-Vicente, Kepa B. Uribe, Shifa Jebari, Unai Galicia-Garcia, Helena Ostolaza, Cesar Martin

**Affiliations:** Departamento de Bioquímica, Instituto Biofisika (UPV/EHU, CSIC), Universidad del País Vasco, Apdo.644, 48080 Bilbao, Spain; asierbenitovicente@gmail.com (A.B.-V.); kepa1985@gmail.com (K.B.U.); shifajebari@gmail.com (S.J.); u.galiciag@gmail.com (U.G.-G.); elenaamaya.ostolaza@ehu.eus (H.O.)

**Keywords:** cholesterol, metabolism, familial hypercholesterolemia

## Abstract

Cholesterol is an essential component of cell barrier formation and signaling transduction involved in many essential physiologic processes. For this reason, cholesterol metabolism must be tightly controlled. Cell cholesterol is mainly acquired from two sources: Dietary cholesterol, which is absorbed in the intestine and, intracellularly synthesized cholesterol that is mainly synthesized in the liver. Once acquired, both are delivered to peripheral tissues in a lipoprotein dependent mechanism. Malfunctioning of cholesterol metabolism is caused by multiple hereditary diseases, including Familial Hypercholesterolemia, Sitosterolemia Type C and Niemann-Pick Type C1. Of these, familial hypercholesterolemia (FH) is a common inherited autosomal co-dominant disorder characterized by high plasma cholesterol levels. Its frequency is estimated to be 1:200 and, if untreated, increases the risk of premature cardiovascular disease. This review aims to summarize the current knowledge on cholesterol metabolism and the relation of FH to cholesterol homeostasis with special focus on the genetics, diagnosis and treatment.

## 1. Cholesterol

Cholesterol was first isolated from gallstones in 1789 during the French Revolution, and since then has been extensively studied. Nowadays, much information about its structure, function and implication in disease development is available [1].

Cholesterol is an essential component of cell barrier formation and cell signaling transduction [2,3] that regulates membrane fluidity and interacts with other lipids and proteins [4]. In addition, cholesterol affects the biophysical properties of the membrane by increasing lipid lateral order and membrane packaging and decreasing membrane fluidity and consequently membrane permeability [5]. Cholesterol can also regulate the function of many proteins, directly by interacting with them [4] or indirectly by its effects on membrane fluidity. Among cholesterol interacting proteins are proteins accepting cholesterol as a substrate (Acyl-CoA acyl-transferase (ACAT)) [6], proteins that need cholesterol-rich environments to effectively interact with the membrane (cholesterol-dependent cytolysins) [7], proteins with sterol binding domains (cleavage-activating protein (SCAP)) or hydroxymethylglutaryl-CoA reductase (HMG-CoA reductase) [8,9], proteins with cholesterol recognition amino acids consensus’ (CRAC) domain and many others [10].

Cholesterol is also the precursor of many steroid molecules as bile salts, steroid hormones and vitamins. Bile salts are synthesized in the liver and are used as highly effective detergents that allow lipid solubilization [11,12]. In the case of hormones, cholesterol is the precursor of five major classes of steroid hormones: Progestagens, glucocorticoids, mineralocorticoids, androgens and estrogens [12]. Vitamin D is also a cholesterol derived molecule with a remarkable importance in calcium and phosphorus metabolism [13].

The complex functions mediated by cholesterol together with its role as precursor and its participation in metabolism pathways require a coordinated input and output regulation to achieve cholesterol homeostasis. This is of significant importance in order to avoid detrimental over-accumulation and abnormal deposition of cholesterol within the body that prevent diseases caused by a failure in cholesterol metabolism.

## 2. Cholesterol Metabolism

### 2.1. Cholesterol Synthesis

Cell cholesterol is mainly acquired from two sources: Dietary cholesterol [14] or intracellular synthesized cholesterol [15]. Almost all tissues have the ability to *de novo* synthesize cholesterol; however, the liver produces the majority of total body cholesterol [16,17]. *De novo* synthesis is a tightly regulated process where several proteins have an important role depending on the specific requirements. Hence, when intracellular cholesterol levels exceed physiologic need, sterol regulatory element-binding proteins (SREBPs) in the endoplasmic reticulum (ER) are inhibited. SREBPs are dedicated sterol sensors in the cell [18] and their activation promotes HMG-CoA reductase transcription (the limiting enzyme of the cholesterol synthesis) and concomitantly activates mevalonate (MVA) pathway to increase intracellular cholesterol synthesis. Cholesterol is synthesized in the ER in a 19 step process, then is secreted to the cytoplasm [19] where becomes available and can be distributed or stored as cholesterol esters (CEs) in lipid droplets after its esterification by ACAT [6].

### 2.2. Cholesterol Absorption

**Figure 1:**
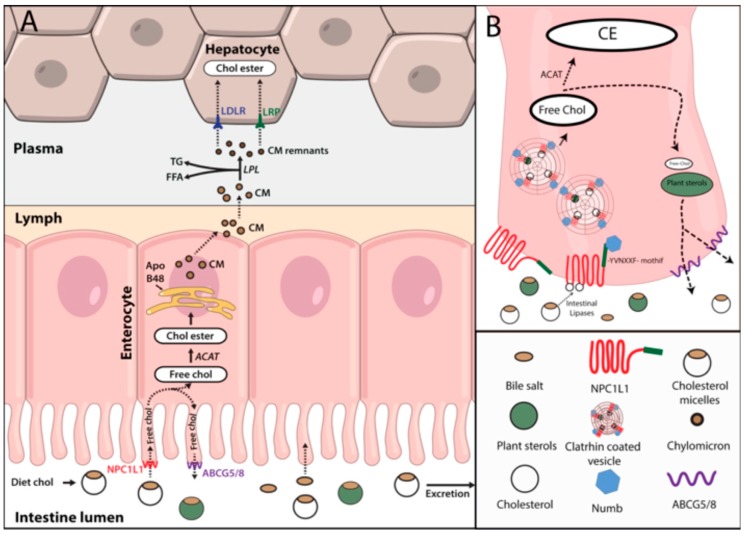
Dietary cholesterol absorption. (**A**) Diet cholesterol forms micelles in complex with bile acids and travel across the intestinal lumen where it is hydrolyzed and taken up by Niemann-Pick C1-like 1 in the enterocyte membrane. Internalized cholesterol can either be transported back to the intestinal lumen through ABCG5/8 along with plant sterols or esterified by Acyl-CoA acyl-transferase. Esterified cholesterol within other lipids is incorporated into chylomicrons and secreted to the lymph. Once in the lymph they are drained to the plasma where by lipoprotein lipases activity lose their triglycerides and become in chylomicron remnants that are finally taken up by the liver by low density lipoprotein receptor or LDLR related proteins. (**B**) Free cholesterol binds NPC1L1 and promotes its conformational change. This conformational change allows the binding of Numb adapter protein to YVNXXF motif and promotes its internalization in clathrin coated pits. Abbreviations: NPC1L1: Niemann-Pick C1-like 1; ACAT: Acyl-CoA acyl-transferase; Chol ester: Esterified cholesterol; CM: Chylomicrons; LPL: lipoprotein lipases; TG: Triglycerides; FFA: Free fatty acids; LDLR: low density lipoprotein receptor; LRPs: LDLR related proteins.

Dietary cholesterol absorption is the second source of cholesterol in the body after *de novo* synthetized cholesterol [20]. Cholesterol, free fatty acids (FFA) and triglycerides are the main lipids coming from the diet and are absorbed in the intestine [21]. Cholesterol absorption by the enterocytes is not an efficient process and for a correct uptake, cholesterol needs to be emulsified by bile acids. Bile acid emulsification generates cholesterol-bile acid micelles that can be delivered to the intestine. There, intestinal lipases hydrolyze cholesterol esters to free cholesterol that is taken up by the enterocytes through Niemann-Pick C1-like 1 (NPC1L1) protein [22]. NPC1L1 has a cholesterol-binding site in its N-terminal domain exposed to the extracellular space and a C-terminal domain bound to the membrane. Free cholesterol interaction with NPC1L1 N-terminal domain, promotes a rearrangement in the intracellular domain of the protein that releases the YVNXXF-containing region from the membrane to the cytosol. Once in the cytosol, Numb, a clathrin adaptor protein, binds and promotes the internalization of the cholesterol-NPC1L1-Numb complex by clathrin coated pits (Figure 1A,B) [22,23].

Once internalized, free cholesterol is delivered to ER where it is either transported back to the intestinal lumen via sterolins (ABCG5/8) or is re-esterified by ACAT. Re-esterified cholesterol can be stored in lipid droplets or directly be packaged together with triglycerides in apolipoprotein B48 (ApoB48) containing lipoproteins (chylomicrons) [24]. Contrary to ACAT, ABCG5/8 have high affinity for plant sterols. Along with ACAT, ABCG5/8 are responsible for the reduced absorption of the plant derived sterols. Indeed, mutations in *ABCG5/8* genes lead to an accumulation of plant sterols in the body, mainly sitosterol, causing a disease condition called sitosterolemia [25].

**Figure 2:**
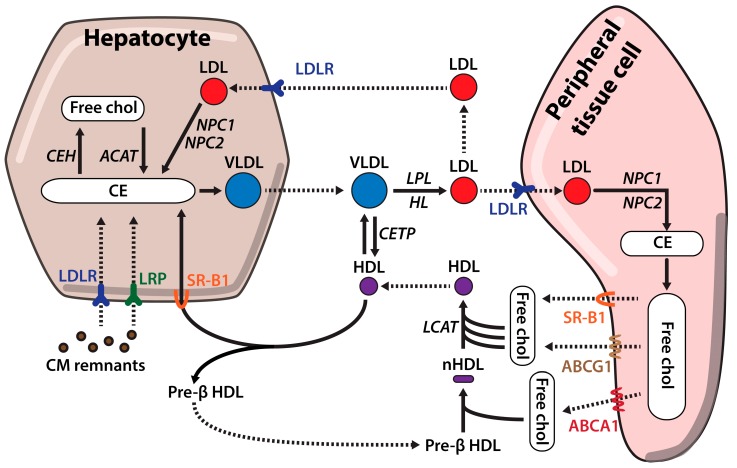
Cholesterol metabolism. Cholesterol is secreted from the liver to peripheral tissues in triglyceride rich lipoproteins, very low density lipoproteins. Once in the bloodstream, VLDL are transformed into cholesterol rich LDL particles by interaction with different proteins as LPL or exchange of lipids and apolipoproteins with high density lipoproteins LDL particles are taken up by peripheral tissue cells through LDLR. Excess cholesterol from peripheral tissues is packaged in HDL lipoproteins for it clearance. First, free cholesterol is transferred to lipid poor pre-β HDL through ABCA1. Second, this first cholesterol loading changes HDL conformation and allow its interaction with ATP-binding cassette subfamily G member 1 and SR-B1 transporters that along with Lecithin-cholesterol acyltransferase produce mature HDL particles that are transported back to the liver for their clearance. Abbreviations: NPC1: Niemann-Pick C1; NPC2: Niemann-Pick C2; ACAT: Acyl-CoA acyl-transferase; CM: Chylomicrons; LPL: lipoprotein lipases; TG: Triglycerides; LDLR: low density lipoprotein receptor; LRPs: LDLR related proteins; VLDL: very low density lipoproteins; HDL: high density lipoproteins; ABCG1: ATP-binding cassette subfamily G member 1; LCAT: Lecithin-cholesterol acyltransferase; HDL: High density lipoproteins.

Chylomicrons are lipoproteins exclusively generated in the intestine during fasting; these particles contain ApoB48, a truncated form of ApoB100 that is produced by an alternative mRNA editing that determines the metabolic role of the chylomicron [26]. In the lipoprotein assembly process is essential the activity of the microsomal triglyceride transfer protein (MTP) [21]. Chylomicrons also contain a large variety of apolipoproteins, such as ApoA-I, ApoA-II, ApoA-IV, ApoA-V, ApoC-I, ApoC-II, ApoC-III or Apo-E, that are incorporated during chylomicron biogenesis or acquired from other circulating lipoproteins [27]. Newly synthesized chylomicrons are secreted into the lymph and transported through the lymphatic system [28] to the thoracic duct where the chylomicron rich lymph is drained into the bloodstream at the left subclavian vein [29]. Then, blood circulating chylomicrons interact with lipoprotein lipases (LPL) of peripheral tissues, primarily adipose and muscle tissue, where LPL is highly expressed [30]. ApoC-II of chylomicrons activates LPL leading to the hydrolysis of triglycerides [31,32]. The released FFAs are actively taken up by adipocytes and muscle cells through fatty acid transporters and CD36. Hydrolysis of FFAs from chylomicrons results in smaller particles enriched in cholesterol esters that transfer ApoA and ApoC to other lipoproteins (basically high density lipoproteins (HDL)) and acquire ApoE [33]. Finally, chylomicron remnants are cleared from the plasma by the liver, due to interaction of ApoE with low density lipoprotein receptor (LDLR) and other LDLR related proteins (LRPs) (Figure 2) [34].

### 2.3. Hepatic Cholesterol Efflux

The liver is the primary organ regulating cholesterol homeostasis and plays a key role in cholesterol synthesis [16], lipoprotein synthesis and secretion [35], lipoprotein clearance [36] and cholesterol excretion among other processes [37]. Cholesterol is secreted from the liver in triglyceride rich lipoproteins known as very low density lipoproteins (VLDL). Regulation of VLDL synthesis and secretion is extremely well coordinated as they are critical for cholesterol distribution. VLDL synthesis is a two-step process that starts with the translocation of nascent apoB100 across the ER membrane of the hepatocytes and becomes lipidated by MTP [38]. If ApoB100 is not well lipidated, due to low triglyceride concentration or a failure in the process, the apolipoprotein is directed for degradation [39]. In a second step, partially lipidated VLDL particles are transported to the Golgi in vesicles containing coat protein complex II (COPII) [40]. Once in the Golgi they acquire apoA1 and apoE apolipoproteins and get further lapidated [41]. Finally, mature VLDL particles are secreted into the bloodstream and transport lipids to peripheral tissues [42].

High level secretion of VLDL by the liver can eventually be translated into high low density lipoprotein (LDL) levels in plasma and an enhanced cardiovascular risk [43]. On the other hand, an impaired VLDL secretion leads to lipid accumulation in the liver, which can be the starting step of fatty liver disease, therefore both processes need to be well coordinated and regulated [43,44].

### 2.4. Cholesterol Influx

**Figure 3:**
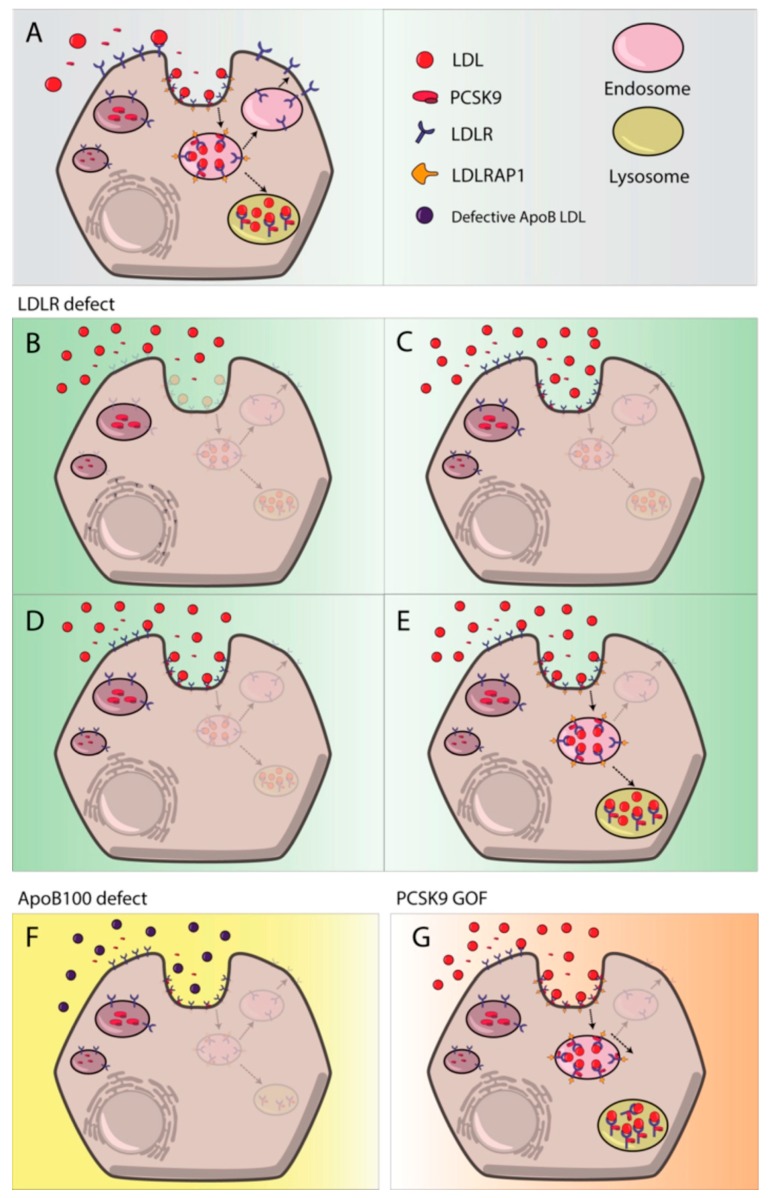
Most frequent LDL catabolism defects. (**A**) LDL uptake process by LDLR; (**B**) class 2 LDLR mutants, LDLR retention in the endoplasmic reticulum; (**C**) Class 3 mutants, no LDL-LDLR binding; (**D**) class 4 mutants, impaired LDL-LDLR complex internalization; (**E**) class 5 mutants, recycling defect; (**F**) defective ApoB-100 derived impaired LDL-LDLR binding; (**G**) PCSK9 gain of function mutant.

Apart from *de novo* synthesized cholesterol, cells obtain cholesterol from the uptake of plasma lipoproteins through LDL receptor (LDLR) pathway [4]. In plasma, triglycerides from VLDL are removed by the action of LPLs and produce VLDL remnants also known as intermediate density lipoproteins (IDL). Additional IDLs processing by hepatic lipases (HL) together with exchange of lipids and apolipoproteins with HDL leads to low density lipoproteins (LDL) formation. LDLs are mainly composed by cholesterol esters and apoB-100 and they are the main cholesterol carriers of the body. They deliver cholesterol from liver to peripheral tissues where they bind LDLR and are endocytosed in clathrin coated pits [45]. Once LDL binds LDLR, LDL receptor adaptor protein 1 (LDLRAP1) recognizes the NPXY motif in the cytoplasmic tail of the LDLR and allows clustering of LDLR into clathrin-coated pits [46]. LDLR-LDL complex is delivered to endocytic compartment where, due to pH acidification, LDLR dissociates from LDL and is recycled back to the membrane due to pH dependent conformational change (Figure 3A). Dissociation of LDL from LDLR in the endosome is a key process that enables receptor recycling while LDL-cholesterol is hydrolyzed in the lysosome, because lysosomal lipase action to release free cholesterol [47,48,49]. Finally, free cholesterol is transferred from lysosomes to the ER by the action of Niemann-Pick type C1/C2 (NPC1/NPC2) proteins [50].

#### Cholesterol Influx Regulation

LDLR mediated cholesterol internalization is a tightly regulated process with several checkpoints both at the transcriptional and post-transcriptional level [18,51,52]. On one hand SREBP-2, an inactive sterol regulatory element located in the ER, is activated at high intracellular cholesterol levels and translocates into the nucleus thus promoting *LDLR* transcription [18,53]. On the other hand, proprotein convertase subtilisin/kexin 9 (PCSK9) [54] and IDOL, an inducible degrader of the LDLR, regulate LDLR at the membrane level by impairing the recycling of LDLR and promoting its degradation [55].

PCSK9 is the ninth member of the protein convertase family that is synthesized as a proprotein and requires an autocatalytic cleavage to become mature [56]. The result of the cleave is a N-terminal prodomain that remains bound to the catalytic domain and inhibits convertase function [57]. Mature PCSK9 is secreted to the extracellular medium where it binds epidermal growth factor A (EGF-A) domain of the LDLR and the complex is internalized by clathrin-mediated endocytosis. Upon endosome acidification, affinity of PCSK9 to LDLR increases thus impairing the required conformational change of LDLR for recycling. The non-dissociated PCSK9-LDLR complex is then delivered to the lysosome [58,59].

IDOL, like PCSK9, regulates LDLR pathway post-transcriptionally. IDOL is an E3 ubiquitin ligase that mediates ubiquitination and degradation of LDLR [60]. IDOL expression is induced by oxysterols, generated due to high intracellular cholesterol concentration; activated cholesterol sensing nuclear receptors (LXRs) enhances IDOL expression along with genes involved in reverse cholesterol transport (RCT) [61]. The up-regulation of IDOL promotes ubiquitination of the cytoplasmic tail of the LDLR and its subsequent degradation [51,60].

Additionally, epigenetics and post-translational modifications of LDLR are important for LDLR mediated lipoprotein uptake [62,63,64]. For instance, microRNAs (miRNAs) have an active role in modulating efficiently LDLR expression. miRNAs are small single stranded non-coding RNAs with the ability to inhibit mRNA translation. They are synthesized as 70–100 nucleotide pri-miRNA, which are sequentially modified by Drosha and Dicer endonucleases to produce mature miRNAs [65]. The mature miRNAs are about 20 nt long molecules that are incorporated into RNA-induced silencing complex (RISC) to target mRNAs for their translational repression [66]. Many studies have shown that miRNAs are important regulators of the LDLR dependent LDL uptake. Indeed, miR-27b [67], miR-27a [68], miR-148a [69] and miR-128-1 [64] among other miRNAs are able to directly bind the 3′UTR of the LDLR mRNA and selectively degrade it.

### 2.5. Reverse Cholesterol Transport (RCT)

RCT is a tightly controlled mechanism by which the body is able to excrete the excess of cholesterol from peripheral tissues through the liver to the feces [70]. In this process, ApoA-1 containing HDL are the mayor cholesterol acceptor from extra-hepatic tissues and the main responsible for cholesterol excess clearance [71].

RCT begins by interaction of ApoA-1 lipid free particles (pre-βHDL) with the ATP-binding cassette transporter A1 (ABCA1) in the plasma membrane that promotes free-cholesterol efflux from the endocytic compartment [71]. Lipidation of pre-βHDL induces conformational changes within the lipoprotein that adopts a discoid shape thus becoming a nascent-HDL. Free cholesterol is then esterified by Lecithin-cholesterol acyltransferase (LCAT) generating spherical mature HDL with a CE core [72]. In addition to ABCA1, the mature HDL can interact with other cholesterol transporters as ATP-binding cassette subfamily G member 1 (ABCG1) or Scavenger receptor class B type 1 (SR-B1) enhancing free-cholesterol efflux from peripheral tissues and increasing HDL particles size [36,72,73]. Passive cholesterol transport from cell membranes to nascent HDL also contributes to cholesterol loading of the lipoprotein [74]. Once HDL are fully lipidated, they are transported to the liver where CEs are selectively removed by SR-B1 for their excretion into bile (Figure 2) [36].

Interactions between different lipoproteins are also common during RCT. Mature HDL exchange both, proteins and lipids with other lipoproteins in plasma. CEs are transferred from the core of the mature HDL to VLDL by the action of cholesterol ester transfer protein (CETP) and receives triglycerides in exchange [75]. As a consequence, VLDL remnants are generated and converted to LDL that can either be removed by LDLR in extra-hepatic tissues or by the liver for excretion into bile [76].

### 2.6. Bile Acid Excretion

Cholesterol excretion into bile is the last step in cholesterol elimination [76]. Bile acids are key modulators of cholesterol homeostasis and the main component of the bile. As mentioned before, they are essential for diet cholesterol emulsification and absorption and they also participate in the excretion of cholesterol leftovers from the liver [77]. Bile acids are primarily synthesized from cholesterol in the liver through a complex pathway strongly regulated by a feedback mechanism [78]. Once synthesized, they are pumped out by ABCB11 (also known as bile salt export pump (BSEP)) and promote secretion of phospholipids and cholesterol to the canalicular plasma, leading to micelle formation [37]. Cholesterol transport to the bile is mainly enhanced by ABCG5/8 heterodimer [79,80] and requires phospholipids because they are a critical component for micelle formation. Indeed, defective phospholipid transport by ABCB4 (historically named multi-drug resistance P-glycoprotein 2(MDR2)) almost completely eliminates cholesterol secretion [81]. Finally the micelles are stored within the gallbladder and released into the intestinal lumen after food ingestion-produced stimuli [82].

In summary, cholesterol metabolism is a complex mechanism with many factors involved that requires a high level of coordination. Cholesterol absorption in the enterocytes, lipoprotein transport, cholesterol uptake in peripheral tissues and cholesterol excretion in the liver are tightly controlled processes that allow a correct balance of cholesterol in the organism. Hence, deregulation of these processes or mutation affecting proteins involved in these pathways can be disease causing. Mutations that alter LDL metabolism are the most frequent defects leading to a cholesterol metabolism derived disease denominated familial hypercholesterolemia.

## 3. Familial Hypercholesterolemia

Familial hypercholesterolemia (FH) is a common inherited autosomal co-dominant disorder primarily characterized by high plasma levels of low-density lipoprotein cholesterol (LDL-C), due to its reduced catabolism [14]. If untreated, exposure to high LDL-C levels during lifetime increases atherosclerotic plaque development and premature cardiovascular disease risk [83].

FH prevalence in its heterozygous form (HeFH) has traditionally been considered to be approximately 1:500. However, frequency can vary between 1:200 and 1:300 depending on which criteria are used to define FH (mutation only, LDL-C threshold only, clinical score or a combination of factors) and the studied populations [84]. Regarding the homozygous form of the disease (HoFH), the prevalence has traditionally been estimated in 1:1,000,000, but recent studies have revealed a prevalence upwards to 1 in 300,000 [84].

### 3.1. Genetics of FH

Cholesterol metabolism and its distribution is a complex system in which many proteins and pathways are involved. LDL catabolism is one of the key points in this process and any defect in its function by any of the proteins taking part on it can generate FH. The major determinants in that system are *LDLR*, accounting for 80–85% of FH cases, *apoB100*, causing 5–10% of the cases, *PCSK9* 2% of the cases and *LDL receptor adaptor protein 1* (*LDLRAP1*) accounting for less than 1% of the cases [85]. Mutations in *APOE* [86], *signal transducing adaptor family member 1* (*STAP1*) [87], *lysosomal acid lipase* (*LIPA*) [47], *ABCG5* or *ABCG8* [88] genes can also generate a FH like phenotype, but its frequency is very low in all of the cases.

#### 3.1.1. *LDLR*

*LDLR* with more than 3000 variants already reported (Clin Var database [89]) is one of the key genes responsible of FH development [49]. LDLR removes LDL from plasma circulation (Figure 3A) and malfunctioning of LDLR is commonly associated with high levels of circulating LDL-C. Many different *LDLR* variants have been described as pathogenic, including large-scale DNA copy number variation (CNV), insertion and deletions, nonsense and missense mutations and splicing mutations [85,90,91]. CNV, nonsense and splicing mutations are commonly associated with higher LDL-C levels [49,92,93,94] than missense mutations. LDLR mutations can affect at different steps of the LDL uptake system and thus can be classified depending on their phenotypic behavior as: Class 1 mutants are characterized by a null protein synthesis; class 2 mutants are partially or completely retained in the endoplasmic reticulum (Figure 3B); class 3 mutants have a binding defect and are not able to properly interact with apoB apolipoprotein (Figure 3C); class 4 mutants have an impaired endocytosis (Figure 3D) and finally class 5 mutants affect the recycle mechanism and LDLR cannot be recycle back to the membrane (Figure 3E).

#### 3.1.2. *APOB*

Mutations in *APOB* are a second cause of FH with a phenotype known as familial defective APOB [85]. Mutations in *APOB* gene were first detected in the highly conserved receptor binding-site (exons 26 and 29) [95] leading to deficient binding to LDLR. Recently some studies have also described new variants out from the consensus binding site of the *APOB* [96], these variants have been functionally characterized and classified as pathogenic indicating that LDLR-LDL binding could be more dynamic than expected [48]. *APOB* pathogenic variants are associated with lower LDL-C levels than those observed with *LDLR* pathogenic variants (Figure 3F).

#### 3.1.3. *PCSK9*

*PCSK9* variants started to be described in the early 2000s when PCSK9 locus was mapped [54]. These variants can either be loss of function (LOF) variants, generating less functional proteins or gain of function variants (GOF) producing more active proteins [97]. GOF variants are associated with increased LDL-C levels as they enhances degradation of LDLR extracellularly, due to increased affinity (Figure 3G) or intracellularly while it is been transported to the membrane [98]. Both mechanisms lead to a reduced expression of LDLR resulting in plasma LDL accumulation. To date, more than 30 GOF PCSK9 variants have been reported; most of them missense mutations located all around the 3 domains of PCSK9 [97]. Different mechanisms underlying the increased activity, including increased transcription, altered autocatalysis or enhanced binding ability for the receptor have been described [97]. LOF mutations are less common than GOF mutations and are associated with lower LDL-C levels and reduced cardiovascular disease [99].

#### 3.1.4. *LDLRAP1*

*LDLRAP1* mutations constitute the fourth most common protein defects among LDLR cycle proteins and cause autosomal recessive hypercholesterolaemia [100]. Pathogenic mutations in both alleles of the gene impair LDLR-LDL complex internalization. A dysfunctional LDLRAP1 does not allow proper clathrin coated endosome formation and inhibits LDL uptake thus increasing plasma LDL-C accumulation [101,102].

### 3.2. Second Generation FH

High cholesterol levels are frequently associated to genes or processes related with cholesterol trafficking, but sometimes they can be a consequence of other diseases or environmental factors. Mutations in *ABCG5* or *ABCG8* genes cause sitosterolemia, in which patients present increased LDL-C levels, some characteristic FH phenotype features and higher cardiovascular risk, alike FH [103,104]. The main cause of this manifestation is plant-sterol accumulation, therefore its treatment consist sterol absorption inhibitors administration instead of statin treatment [25]. Nephrotic syndrome (NS) is also associated with an increased cholesterol and triglyceride accumulation. Patients with acute NS have marked proteinuria that generates an increased synthesis of lipoproteins in the liver [105,106]. Liver failure [107], hypothyroidism [108] or cholestasis are other diseases associated with higher levels of plasma cholesterol and an increased risk of cardiovascular disease [109].

### 3.3. FH Implication in Cardiovascular Disease

**Figure 4:**
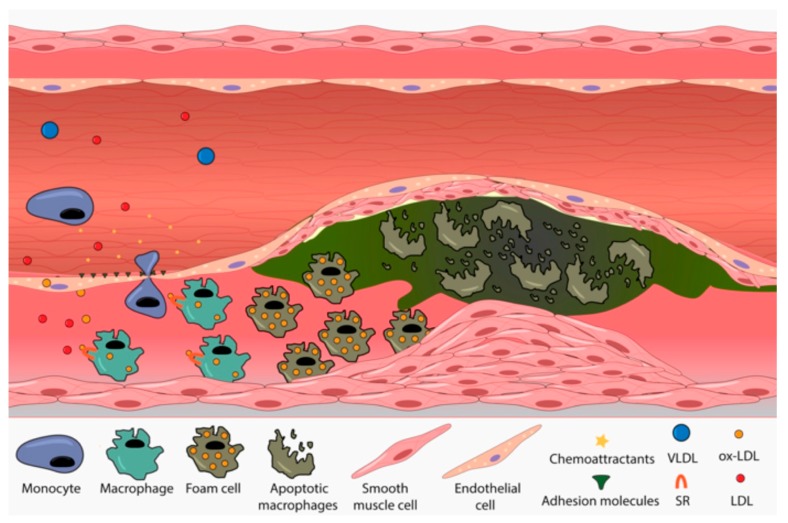
Atherome plaque development. Accumulated LDL particles cross endothelial barrier and get oxidized in subendothelial space. Lipoprotein oxidation activates endothelial cells that increase the synthesis and secretion of chemoattractants and adhesion molecules promoting monocyte recruitment and transedothelial migration. Once at sub-endothelial compartments they are differentiated into machrophages and start to internalize ox-LDL through a non regulated SR-B1 scavenger receptor. Cholesterol excess in macrophages induces foam cell formation and promotes SVMC migration and fibrous cap synthesis. Finally, due to increased macrophage death and impaired efferocytosis, the size of the plaque increases and the diameter of the artery is reduced.

FH is characterized by abnormally increased levels of LDL-C, which promotes early atherosclerosis development [84]. Atherosclerosis is the underlying cause of cardiovascular disease, increasing the risk of heart attack, stroke and peripheral vascular disease [110]. In FH patients, accumulated plasma LDL-C particles and VLDL remnants cross the endothelial lining of the arteries and get retained in the subendothelial compartment where they become oxidized [111]. This lipid accumulation is enhanced in places where the endoplasmic barrier and the junction between endothelial cells are weaker, mainly in arterial curves and branches where a disturbed blood flow causes higher stress [112]. Endothelial cells increase the secretion of different adhesion and chemoattractant molecules in response to oxidized particle stimuli thus generating monocyte recruitment and trans-endothelial cell migration [113]. Once in the subendothelial space, monocytes differentiate to proinflamatory macrophages and start to internalize modified lipoproteins through a non-regulated variety of scavenger receptors (SRs) [114]. The excess of cholesterol in macrophages induces foam cell formation and enhances their proinflamatory fate by promoting migration of vascular smooth muscle cells (VSMC) and proliferation of those macrophages already present in the intima. Finally, activated VSMC start the production of the fibrous cap, an extracellular matrix composed primarily of collagen, elastin and proteoglycans [115]. As atherosclerosis progresses, the necrotic core, covered by the fibrous cap, increases in size as a consequence of increased macrophage death and impaired efferocytosis (Figure 4). This process reduces the diameter of the arteries causing lumen occlusion; furthermore, at this stage, proinflamatory cells present in the plaque start to secrete matrix metalloproteinases (MMPs) that degrade the extracellular matrix of the fibrous cap. This process promotes plaque rupture ensuing formation of thrombi by platelet aggregation and ischemic event thus increasing the risk heart attack and peripheral vascular disease [116,117].

### 3.4. FH Diagnosis

Different guidelines are available for FH diagnosis. Among them, Simon Broome Register Group (SBRG) [118], Make Early Diagnosis to Prevent Early Death (MEDPED) [119] and Duch Lipid Clinic Network (DLCM) [85] are the most extended ones. All the guidelines share common criteria with small differences in the threshold and, combination of factors needed for a definitive FH diagnosis, including physical symptoms (tendinous xanthomata and arcus cornealis if under 45 years old), plasma cholesterol levels (if are over 330 mg/dL is a definite diagnose), familial history of FH, clinical history of the patient and DNA analysis.

### 3.5. Evolution of Lipid Lowering Therapies

High cholesterol levels do not have any direct symptoms so many people usually ignore that they suffer FH. Typically, 200 mg/dL total cholesterol and 100 mg/dL LDL-C are considered threshold values over from which the risk of suffering CVD increases dramatically [120]. Therefore, prevention and treatment of FH is critical. Nowadays, statins constitute the gold standard treatment of FH, but historically other drugs or drug-combination have been commonly used. Additionally, statin treatment in some cases of HeFH and in HoFH only show promising results if combined with other cholesterol lowering therapies [121,122].

#### 3.5.1. Statins

Statins are the most frequently prescribed blood-lipid lowering drugs in the world. They inhibit HMG-CoA reductase and the downstream metabolite production of mevalonate pathway, which is a key step on the production of cholesterol in the liver [1,2,3]. As a consequence, intracellular cholesterol production highly decreases thereby inducing LDLR expression on the hepatocyte cell membrane leading to decreased circulating LDL-C concentrations. Indirectly, statins increase LDL and even VLDL clearance from the plasma, due to overexpression of LDLR in the liver and peripheral tissues [123]. In addition to this, statins also have beneficial effects on other lipid parameters, including increases in HDL levels and decreases in triglyceride concentration. Statins were discovered in the early 70s by Akira Endo, but not commercially available until 1986 when lovastatin was commercialized as the first HMG-CoA reductase inhibitor [123]. Currently, the most frequently used statins are lovastatin, fluvastatin, atorvastatin, sinvastatin and rosuvastatin. The most potent ones: Rosuvastatin, atorvastatin and sinvastatin can also reduce LDL levels in HoFH patients probably because of lower LDL-C production in the liver [124].

Statins can be grouped in two types. Type 1 statins, derived from natural sources or modifications of natural molecules (lovastatin, simvastatin, mevastatin and pravastatin); and, type 2 statins that are synthetic in which typically a fluorophenyl group substitutes the butyrl group present in type 1 statins (atorvastatin, fluvastatin, rosuvastatin, cerivastatin and pitavastatin). Statin hydrophilicity is determinant for their hepatoselectivity. Acording to it, they can be classified into two categories: Hydrophilic (rosuvastatin and pravastatin) and hydrophobic (atorvastatin, simvastatin, fluvastatin, lovastatin and cerivastatin) [125,126]. Both categories are selectively absorbed in hepatocytes however, they show differential absorption in peripheral tissues [127,128]. Hydrophobic statins tend to have higher exposure in non-hepatic tissues because they can passively diffuse through cell membranes whereas hydrophilic statins are more liver specific because they use active transporters to be taken up by hepatocytes. Differences in the differential metabolism of lipophilic and hydrophilic statins provide a mechanism underlying the adverse metabolic consequences. Lipophilic statins are metabolized via cytochrome P450 (CYP450 family of enzymes) to a water-soluble form for renal excretion. In contrast, the water-soluble statins depends less or not at all on the CYP450 system and are excreted largely unchanged being less subject to pharmacokinetic interactions.

Although high efficacy and safety of the statins has been demonstrated, long-term high-dose treatments studies have revealed some adverse effects in some individuals. The most common ones are the statin-associated muscle symptoms as muscle pain or weakness [129], therefore water soluble statins (pravastatin, rosuvastatin) are preferred. The influence of statins on the development of type II diabetes mellitus (DMII) is also under study. Indeed, high dose statin treatment has been implicated in the development of DMII [130]. Specifically, lipophilic statins may have adverse metabolic consequences that include impaired insulin secretion and promotion of insulin resistance, whereas water soluble statins are better tolerated. Several adverse effects on hepatic, renal or even cognitive function should not be discarded [131].

#### 3.5.2. Niacin

Niacin, also called vitamin B3 or nicotinic acid, was the first lipid-modifying drug used for the treatment of FH. Niacin reduces FFA mobilization from adipose tissue by inhibiting its protein lipase system. Therefore, the reduced FFA availability in liver impairs synthesis of cholesterol and triglyceride containing particles. The common side effects of niacin are vasodilatation and elevation of hepatic enzymes [132].

#### 3.5.3. Bile Acid Sequestrants

Bile acid sequestrants were introduced in the market in 1975 [133]. These molecules form insoluble complexes with bile acid-cholesterol micelles thus avoiding capture by enterocytes and consequently the micelles are excreted [134]. Because enterocyte-liver cholesterol transit is partially inhibited, liver cholesterol levels are reduced so LDL and VLDL secretion is reduced and consequently their bloodstream concentration [135]. They are proven to reduce both cardiovascular events and derived mortality however; they are usually not well tolerated. Indeed, they interfere with absorption of some fat-soluble vitamins and also with bile acids reabsorption that, in normal condition, are almost entirely reabsorbed. They are not useful in cases of HoFH with null receptor function [136].

#### 3.5.4. Ezetimibe

Ezetimibe is a selective cholesterol absorption inhibitor that blocks the uptake of cholesterol. It inhibits NPC1L1 both at enterocyte lumen and hepatobiliary interface affecting cholesterol, but not trygliceride or fat-soluble vitamin absorption [137]. The inhibition of cholesterol absorption in the intestine results in a reduced chylomicron formation and secretion in addition to bile cholesterol reabsorption inhibition. The sum of these effects leads to a depletion of cholesterol stores in the hepatocytes. Reduced cholesterol content in the liver favors LDLR expression, as well as reduced VLDL generation resulting in lower LDL-C in plasma [138].

#### 3.5.5. Human Monoclonal Anti-PCSK9 Antibodies

Human monoclonal anti-PCSK9 antibodies have been demonstrated to lower LDL-C levels efficiently and reduce CVD especially in high risk patients [139]. Their use is recommended when neither statins nor ezetimide is able to reduce cholesterol under recommended levels [140]. Currently, two different monoclonal antibodies are available, Alirocumab and Evolocumab. Both are Human IgG subtypes that bind circulating PCSK9 and inhibit their binding to LDLR leading to a PCSK9 deficiency-like condition [141]. The absence of functional PCSK9 enhances LDLR recycling and its availability in the membranes thus favoring LDL clearance from the plasma. While anti-PCSK9 antibodies have low side effects and high efficacy in comparison with other drugs, the high cost of the therapy remains a barrier for more widespread implementation of anti-PCSK9 treatments [141].

#### 3.5.6. Other Treatments

Recently some drugs affecting lipoprotein synthesis have been introduced. Lomitapide is a MTP inhibitor, a protein responsible for the assembly of lipids onto the proteins both in hepatocytes and enterocytes. Mipomersen is an antisense oligonucleotide that binds apoB mRNA reducing VLDL and LDL generation in the liver. Both drugs have been associated with many side effects and their use is not recommended except in cases of HoFH or very high cardiovascular risk [142].

Lipoprotein apheresis (LA) is a therapeutic tool normally used in extremely high-risk patients where other therapies have not worked or appear to be not effective. HoFH is a clear example where LA is recommended [143,144]. Usually HoFH patients with no LDLR expression have residual response to statin treatment and monoclonal antibodies against PCSK9 are rarely effective. In those cases, with a really high risk of CVD and poor prognosis LA therapy should be started [143]. LA is only recommended when other lipid lowering drugs are ineffective, due to its high cost and time consuming. In fact, the accessibility to LA is reduced to a few countries [133].

To date, statins are the main used cholesterol-reducing drug, both due to their high efficacy and low cost [140]. They are recommended as first treatment option and only statin-intolerant patients and patients under statin treatment not achieving recommended LDL-C values, the use of other drugs should be suggested. In cases in which statins have low effect, they are usually combined with ezetamibe, PCSK9 inhibitors or both with promising results [140]. In HoFH with non LDLR function, MTP inhibitors or apoB inhibiting oligonucleotides are appropriate the choice to reduce LDLR levels assuming their low tolerability and high cost [141].

### 3.6. Nutraceuticals in FH

Nutraceuticals are natural lipid regulating products recommended in combination with the previously described therapies for different dyslipidemias management [145]. Nutraceuticals are able to affect at different steps of cholesterol metabolism and can improve the effects of the different cholesterol reducing therapies. Plant sterols and green tea are able to reduce dietary cholesterol absorption [146,147], berberine (extracted from a variety of plants) inhibits PCSK9 action [148,149] and monacolins, present in red yeast rice, share structural similarities with statins and inhibit intracellular cholesterol synthesis [150].

### 3.7. Current FH Situation

As mentioned before, untreated FH increases 13 fold the risk of CVD [151]. Sustained high plasma cholesterol levels induces lipoprotein oxidation and infiltration through endothelial barrier enhancing and accelerating progression of atherosclerotic plaque. Nowadays many high efficacy cholesterol reducing therapies are available and their efficacies have been probed [151]. However, the main issue in FH treatment is the underdiagnosis of this disease. In most countries, less than 1% of the population has been diagnosed and only in The Netherlands have diagnosed more than 50% of their population. In Spain for example, the diagnose condition is reduced to the 6% of the population [85,152]. Taking into account that the estimated prevalence of the disease is 1:200–1:300 and global numbers reveal less than 1% of diagnosed population, there are about 24 and 36 million of FH individuals with non definitive FH diagnose and high risk of premature CVD.

Historically, HeFH has been clinically diagnosed based on LDL-C levels, tendon xanthomas, or familial history of coronary artery disease. This kind of diagnosis was able to detect the most severe cases of HeFH and HoFH, but many mild FH phenotypes were not identified. Through improvements in understanding of FH and the development of new generation sequencing techniques, FH mutations causing mild FH phenotypes are now more easily detected. Combinations of both genetic testing and clinical criteria have enabled detection of mild FH phenotype patients and identification of patients with clinical FH and without mutation in FH generating classical genes [152,153].

Differences in LDL-C levels between FH causing mutation carriers with non-carriers vary with age. At young ages, the differences in LDL-C levels are higher than in people over 55 years old, so differences in LDL-C accumulation are set up mainly in early stages of the disease underlying the importance of an early diagnosis and treatment of the FH [154]. Moreover, early treatment of the disease makes possible an efficient low dose statin treatment instead of high dose treatments required in cases where the FH is diagnosed later in life, thus side effects derived from aggressive statin use are avoided [131].

### 3.8. Functional Studies as a Complement to Genetic Testing

Genetic testing is proven to be the best mechanism for a correct early FH diagnosis. However, because the great majority of the variants are not functionally characterized, genetic testing must be complemented to provide an accurate and definitive diagnose [155]. Cosegregation studies, functional studies or a combination of both are good alternatives to complement genetic studies. Cosegregation studies unlike functional studies have the limitation of clinical data availability and alteration carrier number [156]. Functional studies instead can be performed in any research laboratory and not only give information about the pathogenicity, but also about the disease causing mechanism of the different mutations [48,49,157,158].
